# Poor sleep and reduced quality of life were associated with symptom distress in patients receiving maintenance hemodialysis

**DOI:** 10.1186/s12955-016-0531-6

**Published:** 2016-09-08

**Authors:** Raoping Wang, Chunyuan Tang, Xiaofan Chen, Chunping Zhu, Wanna Feng, Pengsheng Li, Ciyong Lu

**Affiliations:** 1 The Blood Purification Center, Department of Nephrology, First Affiliated Hospital, Sun Yat-sen University, No.74 Zhongshan Rd.2, 510080 Guangzhou, Guangdong People’s Republic of China; 2 College of Public Health, Sun-yat Sen University, Guangzhou, Guangdong 510089 People’s Republic of China

**Keywords:** Maintenance hemodialysis, Symptom distress, Quality of life, Quality of sleep

## Abstract

**Background:**

The quality of life in patients receiving chronic hemodialysis is compromised despite of the substantial achievements in treatments. Quality of life in hemodialysis patients have been shown to be associated with decreased survival and increased hospitalization. Therefore, it is necessary to incorporate the managements of symptoms and patient self-perceived well-being as measurements of effective treatments for these patients.

**Methods:**

A survey of symptom distress, quality of sleep and quality of life was performed in 301 maintenance hemodialysis patients using Dialysis Symptom Index, Short Form-36, and Pittsburgh Quality of Sleep Index table. Patients were recruited from five hospitals in Guangdong area of China by convenience sampling.

**Results:**

The prevalence of various symptoms in maintenance hemodialysis patients was between 23.3 and 80.4 %. These patients had compromised sleep and poor quality of life. Moreover, poor quality of sleep and impaired quality of life were associated with high symptom burden of these patients.

**Conclusion:**

The patients receiving chronic hemodialysis generally have heavy symptom distress, which could contribute to the disturbed sleep and impaired quality of life of these patients. Measurements of clinical outcomes for hemodialysis patients should include the management of symptoms and morbidity. The ultimate goal of treatments is to improve patient self-perceived quality of life.

## Background

With the increase in the patients with diabetes and hypertension, the number of patients with end-stage renal disease (ESDR) grows rapidly worldwide [1, 2]. The survival of hemodialysis patients improved due to the advancements in treatments [2]. However, compromised quality of life is common in patients on chronic hemodialysis [3–8]. Notably, reduced life quality was strongly associated with higher risk of death and hospitalization in hemodialysis patients [3, 5–7]. Complex factors contribute to a lower quality of life in hemodialysis patients [6–9]. Among these, poor sleep is common in patients receiving hemodialysis, and studies showed that sleep quality predicts quality of life and mortality risk in haemodialysis patients [9, 10]. Patients with ESRD who are treated with dialysis may experience many threats to their social, physical and mental capacities, both from the symptoms of ESRD, lifestyle alteration and the treatment-related side-effects on daily life. Hemodialysis patients did suffer from a series of physical and emotional symptoms which negatively affect their physical and mental health. However, the association between symptom distress and sleep quality as well as quality of life in maintenance hemodialysis (MHD) patients in China has not been fully addressed. Considering that China has the largest population in the world and there is a rapid growth in the number of hemodialysis patients, it is necessary to explore the symptom burden and its impact on the patient self-perceived well-being of this population in China. Therefore, the goal of this study was to investigate the prevalence of symptoms as well as the correlation between symptom distress and quality of life in MHD patients recruited from five dialysis units in southern China. We hope that the results of the current study would help find solutions to identify potential interventions to prevent adverse outcomes of ESRD patients and to improve their clinical outcomes.

In the present study, we used SF-36 questionnaire to assess the quality of life, the Pittsburgh Seep Quality Index (PSQI) to evaluate the quality of sleep and the Dialysis Symptom Index (DSI) to examine the symptom distress in 301 patients from southern China. The findings of this study provide helpful information for improving current hemodialysis guidelines, as well as choosing optimal treatments to control symptoms, therefore, to enhance their quality of life and clinical outcomes.

## Methods

### Clinical data collection

Patients were recruited from the dialysis unit of five hospitals in Guangdong province, including the First and Second Affiliated Hospitals of Sun Yat-sen University, Hospital of Dongshang District, People’s Hospital of Jiangmen Xinhui City and Huiya Hospital of Huizhou City by convenience sampling. The criteria for recruiting patients were: (1) Symptoms of patients met the requirements for the diagnosis of uremia upon admission to the hemodialysis center, which include: a) history of chronic kidney disease; b) symptoms such as nausea, vomiting, fatigue, anorexia, weight loss, muscle cramps, pruritus and change in mental status; c) estimated glomerular filtration rate (eGFR) < 10 ml/(min · 1.73 m^2^) in patients with non-diabetic kidney diseases; eGFR < 15 ml/(min · 1.73 m^2^) in patients with diabetic kidney disease; (2) Patients should have received at least 3 months of hemodialysis; (3) At the time of survey, patients were under stable conditions without any severe comorbidities of heart, brain and lungs, including chronic or acute heart failure, heart attack, stroke, respiratory failure, acute or chronic liver failure, cancer and dementia; (4) Patients were conscious with normal judgment, were able to understand the questionnaire and willing to participate the survey; (5) Questionnaires were distributed and explained to patients by trained nurses once informed consent was obtained.

Survey was performed by patient self-administered questionnaires. Laboratory tests, quality of life and sleep were evaluated concurrently with the examination of symptoms.

1. General information
  Patients’ general information included gender, age, dialysis modules, dialysis frequency and the duration of dialysis. Results of laboratory tests, including hemoglobin, red blood cell, hematocrit, serum phosphorus, product of calcium and phosphorus and intact parathyroid hormone (iPTH), were also collected at the time of survey. Kt/V, a way of measuring dialysis adequacy, was calculated. In this measurement, K stands for the dialyzer clearance, the rate at which blood passes through the dialyzer, expressed in milliliters per minute (mL/min), t stands for time. Kt represents the volume of fluid completely cleared of urea during a single treatment. V is the volume of water that a patient’s body contains.
2. Symptoms
  The reliability and validity of the Dialysis Symptom Index (DSI) to assess symptom distress in hemodialysis population have been demonstrated in previous studies [11, 12]. We used the DSI to evaluate physical and emotional symptoms as well as their severity. The DSI contains 30 items with each representing a specific physical or emotional symptom. The enrolled patients were asked to report the presence of each symptom at any time during the previous 7 days by answering “yes” or “no”. Using a modified five point Likert scale (0: “not at all bothersome”, 1: “bothers a little bit”, 2: “bothers somewhat”, 3: “bothers quite a bit” and 4: “bothers very much”), the severity of each symptom was assessed by asking patients to rate how much the symptom was bothersome.
3. Quality of Sleep
  The Pittsburgh Sleep Quality Index (PSQI) is an effective instrument used to measure the quality and patterns of sleep in various patients [9, 13, 14], therefore, we chose PSQI to assess the quality of sleep over the last month in our patients. PSQI has 19 self-rated questions and 5 questions rated by partner or roommate. Only self-rated questions are included in the scoring and are used to generate seven composite scores, each of which gives a score of 0–3 points. The results give numbers in seven categories: subjective sleep quality, sleep latency, sleep duration, habitual sleep efficiency, sleep disturbances, use of sleeping medication, and daytime dysfunction. A score of 0 indicates “very good,” 1 indicates “fairly good,” 2 indicates “fairly bad,” and 3 indicates “very bad.” The global score of PSQI is calculated as the sum of the scores from the seven categories, with a range of 0–21. A global score above 7 indicates poor sleep. The PSQI scores from MHD patients were compared to the results of a national norm reported by Liu [15].
4. Quality of Life
  The 36-item short-form has been validated, is used widely across medical disciplines, and can be self-administered by the patients reliably [3, 16, 17]. The SF-36 was chosen to survey health status in the 301 patients on MHD. It is a generic, multi-purpose, short-form health survey with only 36 questions, which measures eight concepts: physical functioning (PF), role limitations due to physical health (RP), bodily pain (BP), general health perceptions (GH), vitality (VT), social functioning (SF), role limitations due to emotional problems (RE), and general mental health (MH). Two summary measures of physical (PCS) and mental (MCS) health are derived from the eight scales. The SF-36 scores of our patients were compared to the level of a sociodemographically equivalent national norm of China [18].

### Statistical analysis

Data were transferred by Epi-data3.1 and analyzed with SPSS13.0 software. Quantitative data was measured by mean and standard deviation, while qualitative data was determined by percentage and proportion. Spearman correlation coefficiency was used to analyze the correlation between quality of sleep and symptom distress, as well as between quality of life and symptom distress. A two-sided test was used to examine all data and *p <* 0.05 was considered statistically significant for all the analyses.

## Results

### General results

The general information of the patients are shown in Table 1. A total of 310 copies of questionnaire were distributed and 301 valid copies were returned. The valid response rate was 97.1 %. There were 177 male (58.80 %) and 124 female (41.20 %). The age of the respondents was 53.5 ± 13.9 years. 81.4 % of the patients received traditional hemodialysis, only 2.3 % were treated with hemodiafiltration and the other 16.3 % of them had to use a combination of both methods. 80.4 % of the patients received dialysis three times per week. The average dialysis duration was 6.26 ± 5.57 years. The laboratory examinations showed that 68.1 % of the patients had a hemoglobin level lower than our laboratory normal standard value (Table 2). 78.7 % of the patients had an increased serum phosphorus level and the iPTH level was elevated in 89.7 % of these patients. Kt/V was above 1.2 in 85.4 % of the patients (Table 2).

**Table 1:** General information of the patients

Variables	Number of patients	Percentage of patients	Mean ± SD
Gender			
Male	177	58.8 %	
Female	124	41.2 %	
Age (years)			53.5 ± 13.9
Dialysis Mode			
Hemodialysis	245	81.4 %	
Hemodiafiltration	7	2.3 %	
Combination of both	49	16.3 %	
Dialysis frequency			
Three times/week	242	80.4 %	
Five times/two weeks	19	6.3 %	
Twice/week	25	8.3 %	
Other	15	5 %	
Average dialysis duration (years)			6.26 ± 5.57

**Table 2:** Laboratory test results of patients on MHD

Parameters		Number of patients (n)	Percentage of patients
Hemoglobin (g/L)	<120	204	68.1 %
	120–160	92	30.6 %
	>160	4	1.3 %
Red blood cell Count (cell/L)	<4 × 10^12	139	46.2 %
	4–5.5 × 10^12	92	30.6 %
	>5.5 × 10^12	70	23.3 %
Hematocrit	<0.420	285	94.7 %
	0.420–0.490	13	4.3 %
	>0.49	3	1.0 %
Serum phosphorus (mmol/L)	<0.97	3	1.0 %
	0.97–1.62	61	20.3 %
	>1.62	237	78.7 %
Product of Calcium and phosphorus	<55	110	36.5 %
	>55	191	63.5 %
iPTH (pg/mL)	<12	5	1.7 %
	12–80	26	8.6 %
	>80	270	89.7 %
Kt/V	≤1.2	44	14.6 %
	>1.2	257	85.4 %

### Symptoms

The patients in this study had a heavy symptom burden (Table 3), 14 out of the 30 symptoms were reported in at least 50 % of the patients. The top three common symptoms were dry skin, itching and difficult asleep, which were reported by 80.4 %, 77.7 % and 76.1 % of the patients, respectively. The three most severe symptoms were itching, decreased interest in sex and difficult asleep with a severity score of 3.41 ± 1.53, 2.83 ± 1.57, 2.70 ± 1.27, respectively (Table 3). Another 10 symptoms were described as at least “bothering somewhat” (Table 3).

**Table 3:** The prevalence of symptoms in MHD Patients

Symptom	Number of patients reported the symptom	Percentage of patients reported the symptom	Severity^a^
Dry skin	242	80.4	(2.79 ± 1.24)
Itching	234	77.7	(3.41 ± 1.53)
Difficulty asleep	229	76.1	(2.70 ± 1.27)
Feel tired or lack of energy	223	74.1	(2.47 ± 1.16)
Trouble falling asleep	206	68.4	(2.57 ± 1.36)
Decrease interest in sex	201	66.8	(2.83 ± 1.57)
Dry mouth	199	66.1	(2.28 ± 1.14)
Difficulty becoming sexually aroused	195	64.8	(2.79 ± 1.60)
Difficulty concentrating	176	58.5	(2.09 ± 1.12)
Bone or joint pain	173	57.5	(2.24 ± 1.28)
Feeling irritable	173	57.5	(2.09 ± 1.18)
Worrying	172	57.1	(2.10 ± 1.16)
Muscle Soreness	161	53.5	(2.02 ± 1.13)
Cough	154	51.2	(1.89 ± 1.03)
Feeling anxious	152	50.5	(1.96 ± 1.16)
Muscle cramps	145	48.2	(1.78 ± 0.96)
Feeling sad	145	48.2	(1.93 ± 1.18)
Headache	144	47.8	(1.83 ± 1.01)
Lightheadedness or dizziness	143	47.5	(1.74 ± 0.92)
Feeling nervous	139	46.2	(1.86 ± 1.11)
Decreased appetite	130	43.2	(1.72 ± 0.94)
Constipation	128	42.5	(1.82 ± 1.11)
Shortness of breath	122	40.5	(1.71 ± 0.97)
Numbness or tingling in feet	117	38.9	(1.72 ± 1.07)
Nausea	106	35.2	(1.52 ± 0.80)
Restless legs/difficulty keeping legs still	95	31.6	(1.57 ± 0.97)
Swelling in legs	94	31.2	(1.51 ± 0.86)
Chest pain	87	28.9	(1.49 ± 0.87)
Vomiting	73	24.3	(1.36 ± 0.71)
Diarrhea	70	23.3	(1.38 ± 0.76)

^a^Based on the five-point Likert scale: 0 “not at all bothersome” to 4 “bothers very much.”

### Quality of sleep

Quality of sleep in our patients was examined using the PSQI. Our results showed that the score of each of the eight categories, as well as the global PSQI score (8.5 ± 4.61 vs 3.88 ± 2.52, *p* < 0.001) of the patients in the current study, was significantly higher than the national norm [15] (Table 4), indicating that the patients had poor sleep.

**Table 4:** The quality of sleep in MHD patients

Components of PSQI	Patients (*n* = 301)	National norm^a^ (*n* = 112)	*p*
Sleep quality	1.46 ± 0.85	0.63 ± 0.68	<0.001
Sleep latency	1.67 ± 1.1	0.70 ± 0.86	<0.001
Sleep duration	1.68 ± 1.19	0.70 ± 0.58	<0.001
Sleep efficiency	0.44 ± 0.91	0.15 ± 0.47	<0.001
Sleep disturbances	1.14 ± 0.61	0.90 ± 0.44	<0.001
Use of sleep medication	0.7 ± 1.16	0.06 ± 0.24	<0.001
Daytime dysfunction	1.4 ± 0.95	0.73 ± 0.83	<0.001
PQSI global score	8.5 ± 4.61	3.88 ± 2.52	<0.001

^a^Data cited from reference [15]

### Quality of life

Either symptom burden or poor sleep could impair the quality of life; therefore, we measured the quality of life in these MHD patients using the SF-36 questionnaire. Table 5 displayed the SF-36 scores of our patients, compared to the well-accepted national norm-based scores in China. The scores of physical component summary (PCS) and mental component summary (MCS) of the patients were significantly lower than the national norm level of China (PCS: 48.42 ± 21.12 *vs* 77.54 ± 15.96; MCS: 51.64 ± 19.50 *vs* 71.29 ± 17.86; *p* < 0.001). In addition, these patients had a reduced score in each category of the SF-36, compared to the national norm [18]. Overall, the patients had impaired quality of life.

**Table 5:** The quality of Life in MHD patients

Scale of SF-36	Patients (*n* = 301)	National norm^a^(*n* = 17754)	*p*
Physical functioning	61.63 ± 29.20	87.92 ± 16.98	<0.001
Role functioning/physical	31.15 ± 39.94	77.5 ± 34.86	<0.001
Body pain	63.62 ± 22.77	82.22 ± 16.98	<0.001
General health	37.30 ± 18.89	62.51 ± 17.88	<0.001
Vitality	59.67 ± 19.86	68.17 ± 17.63	<0.001
Social functioning	60.47 ± 22.51	80.67 ± 19.98	<0.001
Role functioning/emotional	41.97 ± 45.29	67.86 ± 39.44	<0.001
Mental health	57.29 ± 15.98	68.47 ± 16.90	<0.001
Physical component summary	48.42 ± 21.12	77.54 ± 15.96	<0.001
Mental component summary	54.85 ± 20.13	71.29 ± 17.86	<0.001

^a^Data cited from reference [18]

### Correlation between symptom distress and quality of sleep and life in MHD patients

The impaired quality of life and poor sleep of the MHD patients may be associated with high prevalence of symptoms. To test the hypothesis, Spearman correlation coefficient was used to analyze the correlation between symptom distress and quality of sleep as well as quality of life in these patients. The score of each component of PSQI and the global score of PSQI were positively correlated with both the presence and the severity of symptoms (Table 6) Higher PSQI score, which predicts the poorer sleep quality, was associated with more frequent and worse symptoms. Moreover, a negative correlation between symptom distress and quality of life was revealed by analysis of the Spearman correlation coefficient (Table 6). The score of each component of SF-36 and the global score were inversely correlated with the existence and severity of the symptoms, which suggested that the quality of life in these MHD patients decreased with the increase in symptom burden.

**Table 6:** The correlation of symptom stress with quality of sleep and life in MHD patients

Factors	Presence of symptoms	Symptom severity
	Spearman coefficient	Spearman coefficient
SF-36 Scale		
Physical functioning	−0.383*	−0.478*
Role functioning/physical	−0.326*	−0.382*
Body pain	−0.438*	−0.535*
General health	−0.342*	−0.444*
Vitality	−0.458*	−0.525*
Social functioning	−0.350*	−0.428*
Role functioning/emotional	−0.440*	−0.492*
Mental health	−0.282*	−0.311*
Physical component summary	−0.486*	−0.591*
Mental component summary	−0.511*	−0.584*
SF-36 global score	−0.524*	−0.619*
PQSI components		
Sleep quality	0.430*	0.517*
Sleep latency	0.242*	0.348*
Sleep duration	0.243*	0.305*
Sleep efficiency	0.224*	0.323*
Sleep disturbances	0.410*	0.507*
Use of sleep medication	0.285*	0.362*
Daytime dysfunction	0.558*	0.633*
PSQI global score	0.485*	0.600*

*p < 0.001

## Discussion

The present study revealed that the MHD patients from southern China had a heavy symptom burden. Also, and the high prevalence of symptoms was correlated to a significant reduction in quality of life and sleep. The results of this study suggested that patients on MHD in the current study struggled with a variety of symptoms that may have affected patients’ perception of their position in life and impair physical, social and emotional well-being.

Our findings were largely in agreement with others. Weisbord te. al. carefully examined the association of symptom burden with quality of life in 162 patients [12]. The authors found that four out of the thirty symptoms were reported in at least 50 % of the patients, and overall symptom burden and severity were associated with impaired quality of life [12]. Using the DSI tool, we measured the symptom distress in our MHD patients (Table 3). The patients in our study showed a very similar spectrum of symptoms as those in Weisbord’s study. With the exception of bone/joint pain, the other three most common symptoms reported in MHD patients of Weisbord’s study, dry skin, feeling tired or lack of energy and itching, were also among the most prevalent symptoms in our study (Table 3). Surprisingly, another 10 symptoms presented in over 50 % patients, and even the least common symptom, diarrhea, was reported by 23.3 % of our patients (Table 3). Itching was reported by 77.7 % of the patients and was the most severe symptoms; moreover, another 12 symptoms were ranked as at least “bothers somewhat” (Table 3). It appears that the patients in this study had a heavier symptom burden than the patients in Weisbord’s study [12]. The differences in demographics, ethnicity, culture and socioeconomic background may have an influence in answering the questionnaire, perception and description of the symptoms. However, investigations on MHD patients, albeit of a much smaller scale, from other regions of China disclosed similar pervasiveness and severity of symptoms as ours [19].

The purpose of this study was not set to examine the underlying causes for the symptoms. Nevertheless, we speculated that the high symptom burden reported by the patients in this study may attribute to inadequate dialysis. The biochemical measurements of the patients suggested that the patients might not receive adequate hemodialysis despite that 85.4 % of them had a Kt/V of above 1.2, which is an indicator of small molecule clearance (Table 2). The serum phosphorus levels in 78.7 % of the patients and iPTH levels in 89.7 % of them were higher than our laboratory normal standard (Table 2). Furthermore, 68 % of the patients in this study had a hemoglobin concentration lower than normal which was also an indicator of inadequate renal replacement therapy (Table 2). These results indicated that the dialysis treatment might not be enough in terms of relieving symptoms and removal of phosphates as well as large solutes, such as iPTH. It is possible that non-compliance to diet might have contributed to abnormal biochemical measurements, but we have very clear guidelines for diet control in this patient population (data not shown). We understand that it may not be easy to completely follow the strict guidelines; however, we believe that majority of these patients had good compliance to diet control, based on other hematological measurements, such as serum urea and creatinine concentrations (data not shown).

Symptoms, such as itching, pain, anxiety and worry, can disturb the patients’ day and night rhythm and cause severe sleep problems, which further impair their mental and physical capacity. The high burden of emotional and physical symptoms was significantly correlated to the poor sleep of the patients in the present study (Table 6). The patient with heavier symptom burden generally had worse sleep quality (Table 6). It has been reported that the global sleep quality score was a significant independent predictor of mental and physical health evaluated by SF-36 in hemodialysis patients even after controlling a variety of factors [9]. Similarly, our study not only demonstrated that the MHD patients had reduced life quality, but also disclosed that the quality of life was correlated to the prevalence and severity of symptoms (Table 6). Patients with more as well as severer symptoms reported a poorer quality of life, which was in line with reports by others [9, 10]. The high prevalence and severity of symptoms would have a substantial negative impact on the daily life of patients and could contribute to the poor sleep and impaired quality of life in patients on MHD.

Quality of life is an important measurement of clinical outcomes for patients on hemodialysis [5–7]; specifically, reduced PCS and MCS scores in MHD patients was associated with increased risks in hospitalization and mortality, and increase in PCS and MCS scores predicted better survival [5, 6]. The dialysis clinics in other countries, such as United States, had already included an annual measurement of health-related quality of life in most dialysis patients with the belief that quality of life monitoring has great potential to improve patient outcomes, yielding benefits that exceed burdens for patients and clinics. However, guidelines for adequate hemodialysis in China are mainly based on biochemical measurements and a Kt/V of above 1.2. Kt/V is an index for clearance of small solutes by dialysis. Many studies have demonstrated that there was no association between Kt/V and quality of life or sleep [20–22], indicating that Kt/V may not be an optimal parameter for evaluating dialysis adequacy in regard to improvements in quality of sleep or quality of life. Regardless of a Kt/V of above 1.2 in 85.4 % of these patients, the high pervasiveness of symptoms as well as increases in serum iPTH, phosphorus levels and calcium and phosphorus products suggested that these patients might not receive enough dialysis treatment. Increase in dialysis dose and alternate blood purification methods, such as hemofiltration, hemoperfusion or hemodiafiltration and nocturnal dialysis, which are able to remove uremic toxins more efficiently, may be helpful in improving dialysis effectiveness. Modifications on dialysis regimen have been shown to improve various domains of health-related quality of life [23–25]. Thus, our findings supported the necessity for incorporating evaluation of symptom burden as well as quality of life among patients on dialysis as guidelines for measurements of dialysis adequacy.

Our study also suggested that incorporation of DSI assessment in patient management could provide information for diagnosis of underlying pathophysiological changes and choosing medical treatments of symptoms. For instances, itching, the worst symptom reported in this study, could be resulted from a combination of various factors, such as atrophy of sweat glands, dermal pathological alterations and secondary to hyperparathyroidism [26]. Medical treatment of itching should target these possible factors.

Bodily pain and sexual dysfunction can adversely affect life quality, and depressive symptoms were also associated with morbidity and mortality of patients with ESRD and hemodialysis [12, 14, 27]. Medications and interventions specifically relieving or ameliorating these symptoms could be useful in improving sleep and satisfaction with life in these patients.

We recognized that possible limitations to this study should be addressed. Firstly, the degree that our conclusion can be extrapolated to other areas of China may be considerably limited. However, our results were similar to what had been reported in China [19, 28]; in addition, economy in Guangdong region has been developed better than the majority areas of China. Thus, hemodialysis patients in Guangdong could possibly receive a better socioeconomic support than many other areas in China and we speculated that the nationwide quality of life and clinical outcomes of patients on MHD could be even worse than the results reported in this study. Secondly, we may not be able to fully exclude the patient bias. The patients who were feeling sicker might not be willing to answer the questionnaire, while it was also possible that some patients might have reported severer symptoms in the hopes of obtaining more attention and better treatments from health care givers. Nevertheless, our results were similar to what had been reported both from China and other countries [12, 19, 28], we considered that the patient-bias was minimal. In the end, the quality of life of the patients in this study was evaluated by the SF-36 instrument, which doesn’t take into account of the specific concerns of patients with kidney diseases and ESRD who are treated by dialysis. The effectiveness and reliability of SF-36 in analyzing quality of life have been validated in patients with various diseases, including ESRD and hemodialysis patients [3, 16, 17, 29]. We believe that our results should be able to suggest that quality of life in these patients was generally impaired. Future studies utilizing the Kidney Disease Quality of Life Short Form could facilitate the diagnosis of particular problems associated with kidney diseases, and further provide information for identifying treatments specifically for this population.

## Conclusion

In summary, our results demonstrated that hemodialysis patients from southern China generally had larger symptom distress and higher prevalence of symptoms, compared to patients from better developed countries. More importantly, the compromised quality of life and disturbed sleep in these patients were significantly correlated to the symptom distress. This study suggests measurements of the clinical outcomes for hemodialysis patients should include management of symptoms, morbidity and quality of life. The ultimate goal of treatments for these patients is to reduce hospitalization, increase survival and improve patient self-perceived quality of life.
